# LbCas12a mediated suppression of *Cotton leaf curl Multan virus*

**DOI:** 10.3389/fpls.2023.1233295

**Published:** 2023-08-11

**Authors:** Sidra Ashraf, Aftab Ahmad, Sultan Habibullah Khan, Amer Jamil, Bushra Sadia, Judith K. Brown

**Affiliations:** ^1^Department of Biochemistry, University of Agriculture, Faisalabad, Pakistan; ^2^Cotton Biotechnology Lab, Center for Advanced Studies in Agriculture and Food Security (CASAFS), University of Agriculture, Faisalabad, Pakistan; ^3^Centre of Agricultural Biochemistry and Biotechnology, University of Agriculture, Faisalabad, Pakistan; ^4^School of Plant Sciences, University of Arizona, Tucson, AZ, United States

**Keywords:** agroinfiltration, *Cotton leaf curl virus*, CRISPR/Cas12, plant virus, tissue culture, transgenic plant, virus inhibition

## Abstract

Begomoviruses are contagious and severely affect commercially important fiber and food crops. *Cotton leaf curl Multan virus* (CLCuMuV) is one of the most dominant specie of *Begomovirus* and a major constraint on cotton yield in Pakistan. Currently, the field of plant genome editing is being revolutionized by the CRISPR/Cas system applications such as base editing, prime editing and CRISPR based gene drives. CRISPR/Cas9 system has successfully been used against biotic and abiotic plant stresses with proof-of-concept studies in both model and crop plants. CRISPR/Cas12 and CRISPR/Cas13 have recently been applied in plant sciences for basic and applied research. In this study, we used a novel approach, multiplexed crRNA-based Cas12a toolbox to target the different ORFs of the CLCuMuV genome at multiple sites simultaneously. This method successfully eliminated the symptoms of CLCuMuV in *Nicotiana benthamiana* and *Nicotiana tabacum*. Three individual crRNAs were designed from the CLCuMuV genome, targeting the specific sites of four different ORFs (C1, V1 and overlapping region of C2 and C3). The Cas12a-based construct Cas12a-MV was designed through Golden Gate three-way cloning for precise editing of CLCuMuV genome. Cas12a-MV construct was confirmed through whole genome sequencing using the primers Ubi-intron-F1 and M13-R1. Transient assays were performed in 4 weeks old *Nicotiana benthamiana* plants, through the agroinfiltration method. Sanger sequencing indicated that the Cas12a-MV constructs made a considerable mutations at the target sites of the viral genome. In addition, TIDE analysis of Sanger sequencing results showed the editing efficiency of crRNA1 (21.7%), crRNA2 (24.9%) and crRNA3 (55.6%). Furthermore, the Cas12a-MV construct was stably transformed into *Nicotiana tabacum* through the leaf disc method to evaluate the potential of transgenic plants against CLCuMuV. For transgene analysis, the DNA of transgenic plants of *Nicotiana tabacum* was subjected to PCR to amplify Cas12a genes with specific primers. Infectious clones were agro-inoculated in transgenic and non-transgenic plants (control) for the infectivity assay. The transgenic plants containing Cas12a-MV showed rare symptoms and remained healthy compared to control plants with severe symptoms. The transgenic plants containing Cas12a-MV showed a significant reduction in virus accumulation (0.05) as compared to control plants (1.0). The results demonstrated the potential use of the multiplex LbCas12a system to develop virus resistance in model and crop plants against begomoviruses.

## 1 Introduction

Agriculture is the world’s most important source of revenue and a vital way to improve food security (Dethier and Effenberger, 2012). Improving agricultural productivity through better seeds, irrigation, and other technologies such as precision agriculture and biotechnology can help increase crop yield and reduce the number of people, suffering from hunger and malnutrition. Plant viruses significantly impact food security by causing extensive crop losses, reducing crop yields and crop quality and nutritional value (Makkouk, 2020).

Plant viruses are infectious agents containing genetic material (DNA or RNA) enclosed in a protein coat. They infect and replicate within the cells of plants, causing a wide range of symptoms such as stunted growth, leaf curling and reduced crop yield. Some plant viruses are transmitted by insect vectors, such as aphids, while others may spread through contaminated seeds or mechanical means (Matthews, 2012). Many plant viruses do not have an effective treatment options once infected and control measures are typically focused on preventing infection through crop rotation, sanitation and developing disease resistant plant varieties (Bragard et al., 2013; Panattoni et al., 2013; Sumner, 2018).

There are several families of plant viruses, each with its unique characteristics and replication methods. The most prominent family of pathogenic plant viruses is *Geminivirdae*. Geminiviruses infect economically important crops, ornamental plants, food and weeds, consequently causing extensive yield losses in temperate, subtropical and tropical areas (Zerbini et al., 2017; Al Shihi, 2019). The leading genus of the family *Geminivirdae* is *Begomovirus*, which are characterized with single-stranded DNA (ssDNA) viruses that may be monopartite (2.7 kb) or bipartite (5.4 kb) (Ramesh et al., 2019). *Begomovirus* (genus) currently contains 500 species, including *Cotton leaf curl virus* (CLCuV)*, Tomato yellow leaf curl virus* (TYLCV), *Chili leaf curl virus* (ChiLCV), *Bean yellow mosaic virus* (BYMV) and *Cassava mosaic virus* (CMV). All these viruses cause significant economic losses in various countries by infecting the cash crops such as cotton, tomato, cassava and bean (Kil et al., 2016; Kothandaraman et al., 2016; Zubair et al., 2017b; Thakur et al., 2018). CLCuV is the top-ranked endemic virus specie of *Begomovirus* that infects the cotton crop in Pakistan, India and Africa (Sattar et al., 2013). CLCuV is one of the plant pathogenic virus species that is considered as a primary biotic constraint of cotton yield.

Cotton is an important commercial crop, cultivated annually in many parts of the world. More than 80 countries are cultivating cotton crop, with the major growers are the USA, Pakistan, China, Uzbekistan, and India. The most utilized part of the cotton plant is the cotton bud which acts as an essential raw material to produce many products such as fiber, medicinal products, edible oil, livestock feed and paper (Shan et al., 2014). Cotton is considered as cash crop in Pakistan and the country has consistently ranked among the top cotton producers in the world. The economy of Pakistan gets a substantial boost from its export (6^th^ position in the world ranking as an exporter of cotton). While Pakistan has been a major player in cotton production, it is worth noting that pest attack, drought, and the CLCuV cause a significant loss of yield every year. Over the past three decades, the yield of cotton has been reduced by 30–35% due to CLCuV, which resulted in direct economic consequences for Pakistan (Farooq et al., 2014; Hameed et al., 2014; Rosen et al., 2015; Rehman et al., 2019). The characteristic symptoms of cotton curl leaf disease include vein darkening, leaf curling, enation and vein swelling (Bananej et al., 2016). The most devastating species of this virus is the *Cotton leaf curl Multan virus* (CLCuMuV), a major source of yield reduction in Pakistan and India since the 1980s (Zubair et al., 2017a). CLCuV enters the host plant cell, un-coat and transmits its genome (ssDNA) into the host cell nucleus. Then, the RF gene transforms ssDNA into dsDNA as a replicative form. Bidirectional transcription produces the viral mRNA with the help of host RNA polymerase and viral protein is subsequently translated from viral mRNA. The ssDNA viral genome of CLCuV is replicated and transferred into neighboring cells by plasmodesmata (Yin et al., 2019). A phloem-feeder whitefly (*Bemisia tabaci*) is the main vector that transmits CLCuV from one place to another (Rosen et al., 2015).

Genome editing is a promising tool for overcoming diseases caused through plant viruses by introducing precise changes into the plant genome that confer resistance to the virus. Advancements in genome engineering methods have enabled scientists to introduce the precise gene modifications, i.e., delete, add or replace the genes on the specific target sites in the genomes. These precise genome alterations are preferable substitutes to conventional transgenic approaches (Gao, 2021) to fulfil the early promises of genetic engineering. There are several genome editing tools available, including transcription activator like effector nucleases (TALENs), gene silencing (RNAi), zinc finger nucleases (ZFNs) and CRISPR/Cas (Clustered Regularly Interspaced Short Palindromic Repeats) for installing precise modifications in the genome (Chen et al., 2014; Cheng et al., 2015). Although, all these techniques have their own limitations, such as complex designing and off-target effects (Gaj et al., 2013) but they have succesffuly used to achieve gene editing in bacteria, plants and animals.

CRISPR/Cas system is the most powerful and emerging genome editing technology that is simple, cheap, efficient, and easy to use (Caplan et al., 2015). CRISPR/Cas is a revolutionary technology with enormous applications in bacteria, plants, animals and the medical science as a therapeutic and diagnostic tool (Fellmann et al., 2017; Zhang et al., 2019). Recently, researchers uncovered a new CRISPR-like system in eukaryotes such as OMEGA (Obligate Mobile Element Guided Activity) and Fanzor, that may have broad spectrum applications in eukaryotic gene editing with better efficiency (Altae-Tran et al., 2021; Saito et al., 2023). CRISPR/Cas toolbox have been successfully used to engineer plants with improved biotic and abiotic stresses, enhanced yield and nutritional quality, and herbicide tolerance (Zafar et al., 2020). Similarly, CRISPR system has been effectively employed to achieve different genetic modifications such as gene knockdown, knockout, knock-in, transcriptional regulation through CRISPRi and CRISPRa, and site specific base editing (Malzahn et al., 2017; Yu et al., 2020). It is a straightforward technique and simple techniques, to introduce new traits in plants, without inserting any foreign gene, to generate transgene free CRISPR edited crops with better public acceptance (Ma et al., 2016).

CRISPR/Cas offers a promising approach for controlling plant viruses and producing disease resistant plants to overcome challenges of food security and zero hunger. It is an exciting and rapidly evolving field that holds a great promise for the future of agriculture, health, and sustainable environment. Researchers have demonstrated CRISPR/Cas9 applications, especially multiplex genome editing to introduce precise changes in the plant genomes that prevented the virus from replicating or spreading (Uniyal et al., 2019). Similarly, CRISPR/Cas13 may provide an alternate and better strategy for diagnosis and control of RNA viruses. Therefore, CRISPR/Cas technology offers an efficient and powerful approach to reduce the viral effect of begomoviruses in crop plants (Zaidi et al., 2016). In addition, CRISPR/Cas holds a great potential to enhance the expression of plant defense genes to make the plant more resistant to viral infection (Ali et al., 2015; Baltes et al., 2015; Chaparro-Garcia et al., 2015). For example, CRISPR/Cas was successfully used to improve resistance against several plant viruses including *Tobacco mosaic virus* (Zhang et al., 2018), *Cucumber mosaic virus* (Tzean et al., 2019) and *Potato virus* Y (Hameed et al., 2019). These studies demonstrated the enormous potential of CRISPR/Cas for controlling plant viruses and enhance the productivity of crops.

CRISPR/Cas12 (previously known as Cpf1), belongs to the Class II, type V CRISPR/Cas system, charcaterized from *Acidaminococcus* and *Lachnospiraceae bacterium* (Bernabé-Orts et al., 2019). Cas12 is an RNA-guided nuclease to create sequence specific DSB in the genome, thus allowing targeted DNA modifications. Like other CRISPR/Cas systems, CRISPR/Cas12 can be programmed to target specific DNA sequences and install precise modifications in the genome (Swarts, 2019). CRISPR/Cas12a is an emerging, versatile and powerful genome editing tool for agricultural advancement (Bandyopadhyay et al., 2020).

Compared with CRISPR/Cas9, CRISPR/Cas12 holds several advantages, such as smaller size of Cas12, which allows an efficient delivery into plant cells and its ability to produce sticky-ends (staggered cut), which can simplify the process of integrating edited DNA into the plant genome. In addition, Cas12 requires a T-rich PAM as compared with G-rich PAM in CRISPR/Cas9. T-rich PAM may facilitate the targeting of non-coding regions in the genome though CRISPR/Cas12. These features makes CRISPR/Cas12, a promising tool for genome editing especially in agriculture, health and metabolic engineering. Different subtypes of CRISPR/Cas12 have been used for genome manipulation in living cells (Gosavi et al., 2020). The versatility of CRISPR/Cas12a in plant genome editing has been demonstrated in various studies, where it has been used to edit genes involved in a range of traits including disease resistance (Mishra et al., 2021), abiotic stress tolerance (Rahman et al., 2022) and improved yield. All these applications of CRISPR/Cas12 in plant genome editing demonstrate the great potential of CRISPR/Cas technology, to develop climate resilient and disease resistant crops with better adoptability to climate change and meet the challenges of food security and sustainable development.

## 2 Materials and methods

### 2.1 Virus analysis

DNA sequences of 92 variants of CLCuMuV for period 2019–2021, were collected from the NCBI gene bank and uploaded to the Geneious Prime software. Multiple alignment (Geneious alignment) of 92 variants (period 2019–2021) of the CLCuMuV species was conducted on Geneious Prime software to check the similarity among all these variants. A phylogenetic tree was constructed to evaluate the geographic distribution of these variants on Geneious Prime by using the Geneious Tree builder method. According to geographical distribution, 27 variants of CLCuMuV (present in Pakistan) were selected for analysis.

### 2.2 Designing of crRNAs

Specific sites of open reading frames (ORFs) of CLCuMuV were identified as target sites on Geneious Prime software. crRNAs were designed for CRISPR/Cas12a (LbCpf1) against target sites in the viral genome. CRISPR/Cas12a requires a PAM sequence (TTTN) to target specific regions, therefore, TTTN (N = ATGC) was selected as PAM site for designing crRNA (Doench et al., 2016). Multiple sequences (CRISPR sites) were analyzed on Oligoanalyzer (https://www.idtdna.com/pages/tools/oligoanalyzer, last access: 11-07-2023) and Cas-OFFinder (http://www.rgenome.net/cas-offinder/, last access: 11-07-2023) to select the best crRNAs. OligoAnalyzer is a web tool used to determine the physical characteristics of oligonucleotides. The OligoAnalyzer tool is available online from IDT (Integrated DNA Technologies), a company specializing in oligonucleotide synthesis and related products. The tool allows input of the nucleotide sequence and generates various parameters, such as melting temperature (Tm), GC content and potential hairpin or dimer formation (Jameel et al., 2022). The selected crRNAs were uploaded on the OligoAnalyzer for the analysis of secondary structure, hairpin, self-dimerization and heterodimerization. For this study, the off-target effects of the selected crRNAs were evaluated on Cas-OFFinder software (Bae et al., 2014). Off-target effects for *Nicotiana benthamiana*, *Nicotiana tabacum* and cotton were analyzed. All the properties including Tm, GC content, self-dimerization and heterodimerization were also double-checked on AmplifX software (Franz et al., 2017).

### 2.3 LbCas12a based construct

**Figure 1:**
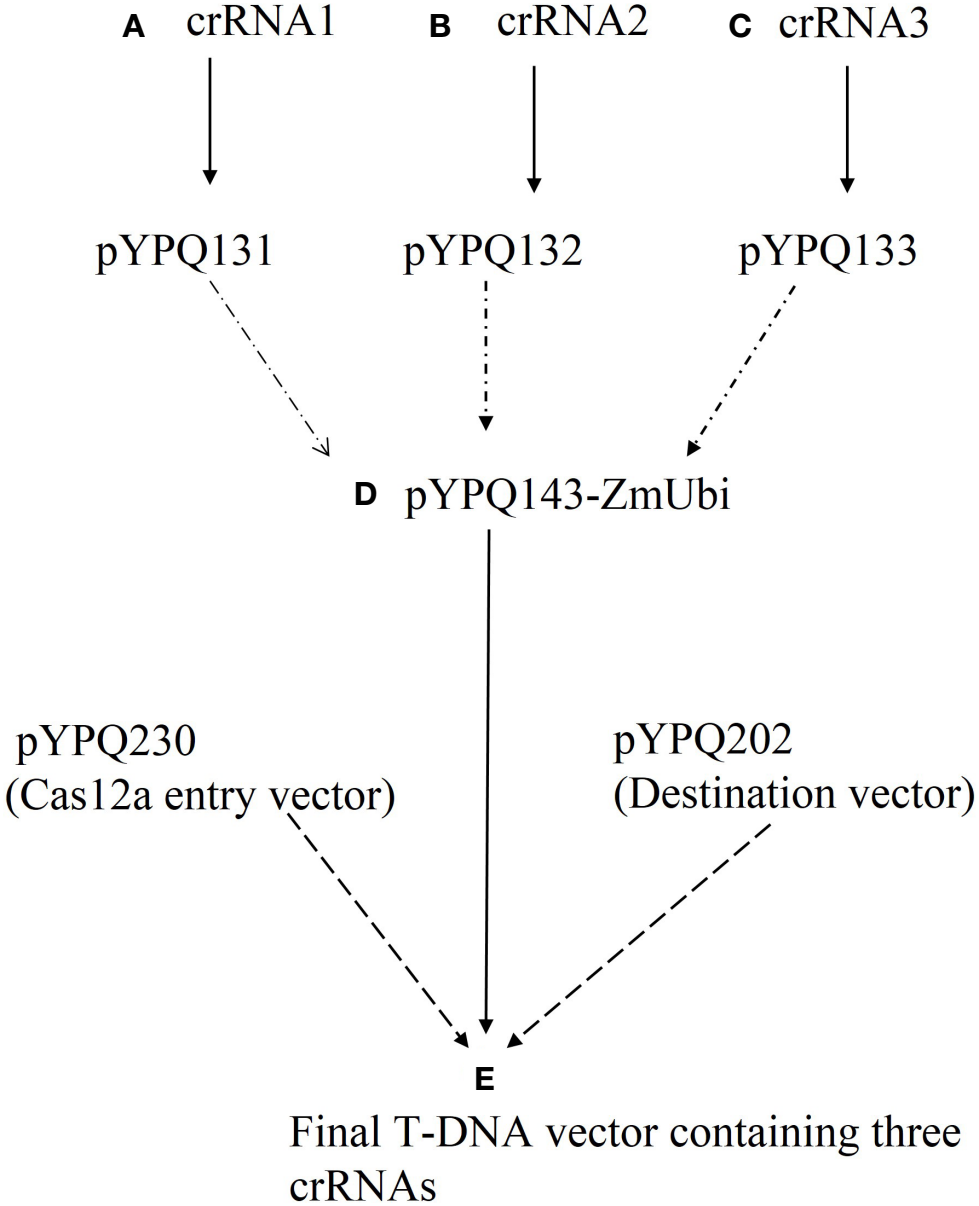
Flow sheet of vector construction with three crRNAs for multiplexed genome editing through CRISPR/Cas12a. **(A)** 1^st^ crRNA was cloned into crRNA cloning vector pYPQ131. **(B)** 2^nd^ crRNA was cloned into crRNA cloning vector pYPQ132. **(C)** 3^rd^ crRNA was cloned into crRNA cloning vector pYPQ133. **(D)** All cloning vectors were assembled in the recipient vector (pYPQ143) using the Golden Gate assembly method. **(E)** Cas12a entry vector (pYPQ230), destination vector (pYPQ202) and crRNA recipient vector (pYPQ143-ZmUbi-pT) were assembled using a three-way Gateway LR reaction to form the final T-DNA vector.

All vectors required to make the construct were obtained from Addgene (www.addgene.org, last access: 11-07-2023) (Zhang and Qi, 2021). The details of plasmids involved in the designing of the T-DNA vector (CRISPR/Cas12a multiplex vector (Cas12a-MV) containing Cas12 and multiple crRNAs) are given in **Table 1**. The T-DNA vector was constructed by the Golden Gate three-way cloning method. Firstly, duplexed oligonucleotides (selected crRNAs sequences) named crRNA1, crRNA2 and crRNA3 were phosphorylated, annealed and cloned into the linearized crRNA expression plasmids such as pYPQ131-STU-Lb (expression vector for crRNA1), pYPQ132-STU-Lb (expression vector for crRNA2) and pYPQ133-STU-Lb (expression vector for crRNA3) at the *Esp3*I restriction site to construct crRNA cassettes. The crRNA cassettes were assembled with respective recipient plasmid (pYPQ143-ZmUbi) to construct crRNA entry vectors through the Golden Gate cloning method. Finally, the crRNA entry vector (cloned pYPQ143), Cas12a entry vector (pYPQ230) and destination vector (pYPQ202) were assembled through a three-way Gateway LR reaction. Flow sheet describing the steps to construct a T-DNA vector (Cas12a-MV) is given in **Figure 1**. The *EcoR*1 restriction enzyme and whole-genome sequencing were used to confirm the successful cloning. **Table S1** shows the list of primers, used for WGS sequencing of Cas12a-MV.

**Table 1:** List of plasmids required to generate Cas12a construct.

Plasmids	Plasmid number	Plasmid name	Purpose	Size	Bacterial selection	Plant selection
crRNA cloning vectors	138096	pYPQ131-STU-Lb	Golden Gate cloning vector for 1st crRNA	3181 bp	Tetracyclin	–
	138099	pYPQ132-STU-Lb	Golden Gate cloning vector for 2nd crRNA	3181 bp	Tetracyclin	–
	138102	pYPQ133-STU-Lb	Golden Gate cloning vector for 3rd crRNA	3181 bp	Tetracyclin	–
Recipient vector	138107	pYPQ143-ZmUbi	Golden Gate recipient; used for the assembly of three crRNAs	5310	Spectinomycin	–
Cas12a entry vector	86210	pYPQ230 (Lb editing)	Cas12a Gateway entry plasmid	6939	Spectinomycin	–
Destination vector	86198	pYPQ202	T-DNA entry plasmid	12807	Kanamycin and Chloramphenicol	Hygromycin

### 2.4 Plant material and transformation

*Nicotiana benthamiana* was used as a model plant to test the potential of the multiplex Cas12a-MV construct. The transformation of plants through agro-infiltration was performed at the School of Plant Sciences, University of Arizona, USA. Seeds of *N. benthamiana* were grown in small pots, containing soil mixed with peat moss at an optimum temperature (28°C) and photoperiods (8 hours dark and 16 hours light photoperiod). After three weeks of transplanting, all the plants reached to an optimal developmental stage indicated by 2–3 fully developed true leaves, with no visible flower buds. These plants were used for infectivity analysis through agroinfiltration (Mubarik et al., 2019). The infectious clones, including *Cotton Leaf Curl Multan Virus- Rajasthan* (CLCuMuV-Ra) and *Cotton leaf curl Multan betasatellite* (CLCuMuB), were used to test the potential of Cas12a-MV construct. Betasatellite infectious clone was used to induce and develop symptoms of CLCuMuV in the plants. Agrobacterium-mediated transient transformation (agroinfiltration) was performed to express the Cas12a-MV construct with CLCuMuV infectious clone in *N. benthamiana*. A total of 36 plants were divided into three groups A, B and C, according to the given treatments. The description of different groups of plants, used in this study is given in **Table 2**.

**Table 2:** Groups of plants according to treatment.

Group	Treatment	No. of plants
Group-A	Negative control (plants containing just inoculation buffer)	12
Group-B	Plants co-infiltered with Cas12a-MV vector and infectious clones	12
Group-C	Positive control (plants inoculated with infectious clones only)	12

### 2.5 Confirmation of viral gene in plants

Genomic DNA of virus was isolated through the CTAB method from all plants after 15 days post-inoculation (dpi) (Healey et al., 2014). PCR was performed using specific primers (according to the viral genome) to check the presence of infectious clones in all plants. The list of primers used in this study to amplify viral DNA from the infectious clones is given in **Table S2**.

### 2.6 Mutation detection

Sanger sequencing was utilized to detect mutation in the CLCuMuV genome isolated from the infected plants. The genomic DNA was isolated from *N. benthamiana* plant leaves after 15 dpi. The target sites were amplified with specific primers through PCR. Purified PCR products were subjected to Sanger sequencing. List of primers used for Sanger sequencing in this study is given in **Table S3**. The resulting traces from Sanger sequencing were uploaded on TIDE software (http://shinyapps.datacurators.nl/tide/, last access: 13-07-2023) and compared with the reference sequence (CLCuMuV genomic sequence) to detect the mutations in the targeted regions. TIDE software uses an R sequencer to analyze the sequencing data. The R Sequencer uses the R programming language, which provides a wide range of statistical and bioinformatics packages for data analysis (Brinkman and van Steensel, 2019). This tool is specific for Cas9 editing but some default settings also make it appropriate for Cas12a editing.

### 2.7 Stable transformation of *Nicotiana* plants with Cas12a construct

Transgenic *Nicotiana tabacum* plants were generated through the leaf disc method (*Agrobacterium*-mediated). The leaves of 4-week-old, wild-type *Nicotiana* plants were picked and surface sterilized with 5% bleach and 0.1% Tween-20 for 5 minutes. In the next step, leaves were washed with sterilized water three times. Leaf discs of 1 cm square in size, were sliced from sterilized leaves and incubated with agrobacterium containing Cas12a-MV construct with an OD 0.8, for 8 minutes at room temperature. Then agrobacterium infected leaf discs were placed on a co-cultivation medium (3% sucrose, 1x MS powder, 1% agar, 2mg/L kinetin, 2mg/L IAA and 200µM acetosyringone) and incubated in the dark for two days at 22°C. After two days, leaf discs were shifted to an induction medium (3% sucrose, 1x MS powder, 1% agar, 2mg/L kinetin, 2mg/L IAA, 200mg/L timentin and 25mg/L hygromycin) and incubated at 28°C for the development of the callus. After a week, the explants were shifted to fresh induction medium until the shooting was initiated. Shoots of 2–3 cm in length were cut from the explant and transferred to the root induction medium (3% sucrose, 1x MS powder, 1% agar, 0.2mg/L IBA, 200mg/L timentin and 25mg/L hygromycin). After three weeks, plants with roots were shifted from rooting medium to peat moss and kept in a plastic box to maintain moisture. Two weeks later, the transgenic plants were shifted to the growth chamber and seeds were collected from mature plants after 4–6 weeks.

### 2.8 Expression analysis of Cas12a and gRNAs in transgenic plants

The transgenic *Nicotiana tabacum* plants were screened through PCR to check the presence of Cas12a. For this purpose, genomic DNA was isolated through the CTAB method from the leaves of *N. tabacum* transgenic plants. Cas12a gene specific primers were used to check the integration of Cas12a in transgenic plants. For expression analysis, total RNA was isolated from putative transgenic plants and used for cDNA synthesis. Real-time PCR was performed to quantify the expression of Cas12a and multiple crRNAs in transgenic plants (Ma et al., 2015).

### 2.9 Virus infectivity assay of transgenic plants

Wild-type and transgenic *Nicotiana* plants (expressing Cas12a and crRNAs) were agro-inoculated with CLCuMuV infectious clones to induce and develop the symptoms of the virus. The virus accumulation in transgenic plants of *N. tabacum* was checked through qPCR and compared with control plants (infiltrated with infectious clones) (Mubarik et al., 2019).

## 3 Results

### 3.1 Target selection

**Figure 2:**
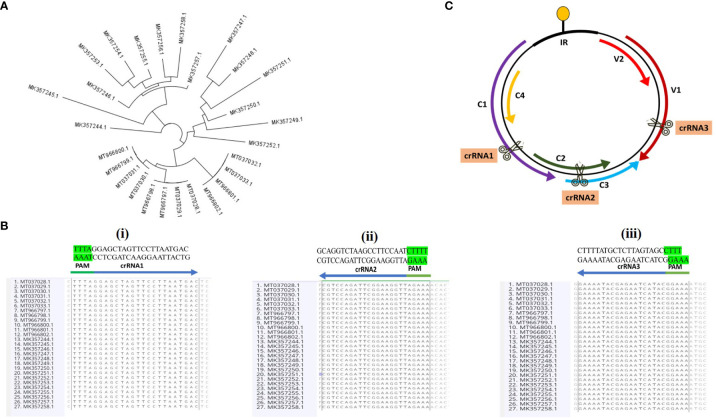
**(A)** The circular phylogenetic layout was derived from the alignments of 27 full-length sequences of CLCuMuV and conducted on Geneious Prime software. It shows the relationship among the different strains of CLCuMuV. The branches of the tree are arranged in a circle around a central point (showing the same ancestor), with the groups of strains placed at the tips of the branches. The length of the branches and the distance between the strains represent the evolutionary distances or differences among them. **(B)** All the nucleotide sequences were retrieved from the NCBI Gene Bank and aligned through Geneious alignment in Geneious Prime software. All the selected crRNAs sequences are shown, and the PAM sequence is highlighted with green color. **(C)** Schematic diagram of the CLCuMuV genome with the target sites. Arrows represent the ORFs (C1, C2, C3, C4, V1 and V2). crRNA1 targets the specific site of C1 and crRNA2 targets the overlapping region of C2 and C3. crRNA3 targets the specific site of V1.

Genomic sequences of CLCuMuV for period 2019–2021 were retrieved from NCBI and analyzed to select the target sites using Geneious Prime software. According to geographical distribution, 27 variants of CLCuMuV were selected with Pakistan origin. The phylogenetic tree of 27 variants is shown in **Figure 2A**. All details of 27 variants including isolate name, accession number, size and origin are given in **Table S4**. The potential consensus sequences in the coding regions were selected as target sites, from 27 species of CLCuMuV, to produce broad-spectrum resistance against virus (**Figure 2B**). Three crRNAs were selected, targeting the C1 region (Rep), overlapping region of C2 (TrAP) and C3 (Ren Protein), and V1 (CP) to inhibit the replication of CLCuMuV (**Figure 2C**). The crRNAs were selected based on different properties including secondary structure, hairpin structure, self-dimerization and heterodimerization. Off-target effects were checked against cotton and *Nicotiana benthamiana*. The detail of selected crRNAs is summarized in **Table S5**.

### 3.2 Plasmid construction

**Figure 3:**
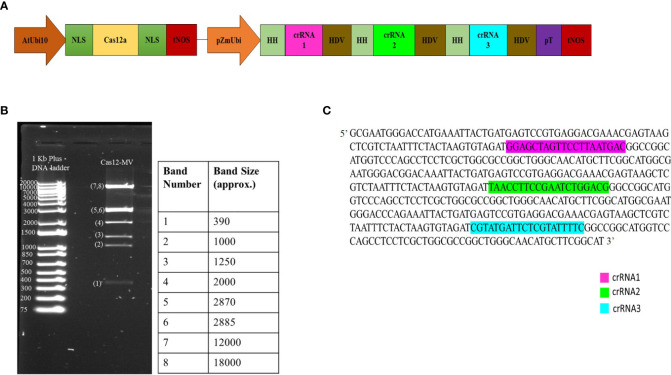
**(A)** Schematic diagram of multiplexed LbCas12a expression cassette with three crRNAs. AtUbi10, *Arabidopsis ubiquitin* 10 promoter; NLS, nuclear localization signal; tNOS, nopaline synthase terminator; pZmUbi, *Zea mays ubiquitin* promoter; HH, hammerhead; HDV, hepatitis delta virus; pT, terminator. **(B)** The construct was confirmed through the restriction enzyme digestion method using *EcoR*I. The product was run on 1.2% agarose gel. 1Kb plus DNA ladder was used (band sizes are mentioned in the table). **(C)** WGS result of the cloned construct of Cas12a-MV verified the presence of all three selected crRNAs (crRNA1, crRNA2 and crRNA3) in the construct.

We designed three crRNAs through Geneious Prime software, targeting the four coding regions (C1, C2, C3 and V1) to impede the replication of CLCuMuV. The crRNAs expression cassette and LbCas12a expression cassette were combined into a T-DNA vector (destination vector) through Golden Gate three-way cloning. In this study, two Pol-II promoters including AtUbi10 and pZmUbi were used to drive expression of Cas12a and tandem HH-crRNA-HDV arrays respectively. crRNAs were flanked with hammerhead (HH) ribozyme RNA and hepatitis delta virus (HDV) ribozyme RNA for precise processing of crRNA. This type of expression is considered as best to derive crRNAs and Cas12a (**Figure 3A**). The cloning vectors including pYPQ131, pYPQ132 and pYPQ133 were digested with the *Esp3*1 enzyme and then crRNA1, crRNA2 and crRNA3 were ligated in them respectively. Next, those multiple cloning vectors were assembled with the recipient vector pYPQ143 by T4 DNA ligase. In the final step, the Gateway LR reaction was done by the assembly of cloned recipient vector, Cas12a entry vector (pYPQ202) and destination vector (pYPQ230) to construct the final T-DNA vector (Cas12a-based construct). The Cas12a-based construct was first confirmed through restriction enzyme *EcoR*1 and resolving its product on agarose gel which showed eight bands with different sizes, as shown in **Figure 3B**. Whole genome sequencing was performed to confirm the cloning of crRNA and Cas12a in the final construct. The specific part of WGS consisting of multiple crRNAs is shown in **Figure 3C**.

### 3.3 Infectivity assay

**Figure 4:**
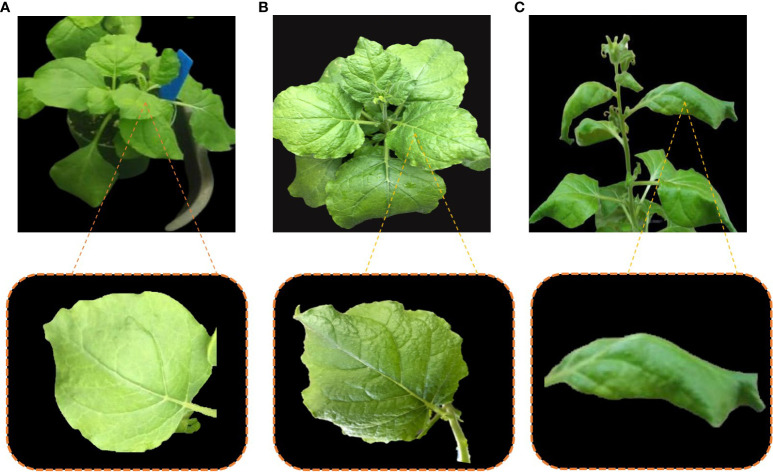
Plants showing susceptibility and resistance against CLCuMuV. **(A)** Plant A is a non-inoculated plant that showed no signs and symptoms of CLCuMuV. **(B)** Plant B is co-infiltrated with the Cas12a-MV construct and infectious clones of CLCuMuV, showed mild symptoms and resistance against CLCuMuV. **(C)** Plant C was inoculated with only infectious clones (CLCuMuV) and showed severe CLCuMuV symptoms.

*N. benthamiana* plants were divided into three groups named as group A, group B and group C. Group A plants were infiltrated with inoculation buffer only and showed no symptoms (**Figure 4A**). Group-B plants were co-infiltrated with infectious clones of CLCuMuV and Cas12a-MV. The appearance of leaf curl symptoms in systematic leaves of group B plants was delayed by 10–12 days as compared with the control plants (**Figure 4B**). The symptoms of the disease were mild, and plants recovered from the disease after 30 days post-infection. Group C plants were kept as positive control and infiltrated only with infectious clones (CLCuMuV). The leaf curling symptoms appeared severe in the group C plants (**Figure 4C**). **Table 3** shows the evaluation of the Cas12a constructs for the suppression of CLCuMuV.

**Table 3:** Evaluation of multiplex CRISPR/Cas12a construct to suppress the CLCuMuV in plants.

Groups	No. of plants	Severity of disease	Symptoms percentage	Recovery of the plant after 30 dpi
A	12	No	0%	–
B	12	Mild	20%	Yes
C	12	Severe	100%	No

### 3.4 Mutation detection and TIDE analysis

**Figure 5:**
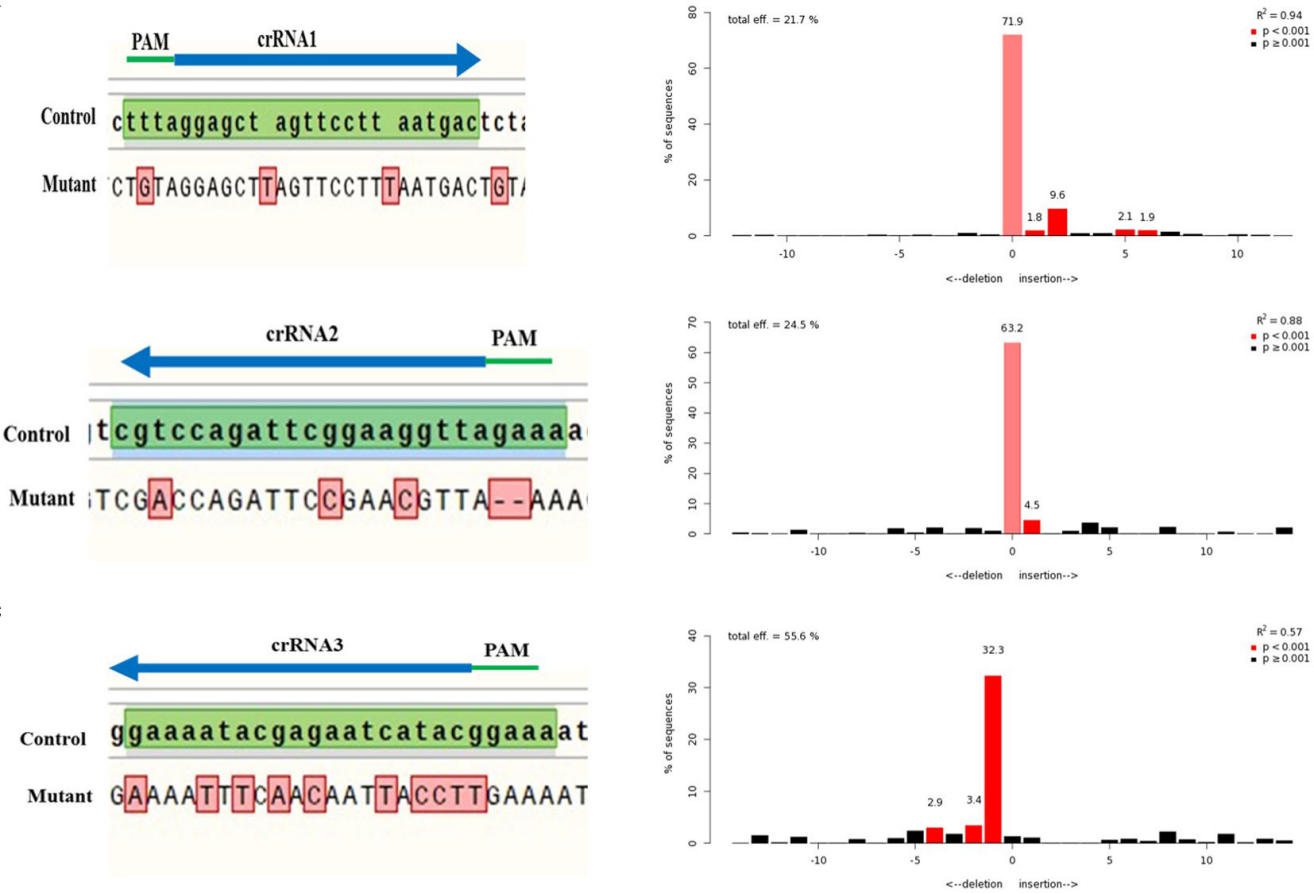
TIDE analysis of CLCuMuV (specific regions) targeted with selected crRNAs. The wild-type sequence of CLCuMuV was used as a control and compared with the targeted mutant sequences. **(A)** crRNA1 targets the C1 region and total editing efficiency of crRNA1 is 21.7%. **(B)** crRNA2 targets the C2 and C3 regions and total editing efficiency of crRNA2 is 24.5%. **(C)** crRNA3 targets the V1 region and total editing efficiency of crRNA1 is 55.6%.

The selected crRNAs in this study, targeted the specific coding sequences of the CLCuMuV genome to reduce its viral effect. Sanger sequencing confirmed the mutations produced at the DSB sites by multiplex CRISPR/Cas12a system. Sanger sequencing is considered as a reliable method for DNA sequencing and detect Cas footprints because it has high accuracy and reproducibility. Comparison of mutant traces and control sequence is shown in **Figure 5**. Recently, an algorithm of TIDE is created to evaluate the Sanger sequencing traces. The trace sequences were analyzed through the TIDE program to access the targeting efficiency of selected crRNAs. This method gives indel spectrum by comparing and decomposing Sanger traces generated from PCR products of edited templates and wild type. TIDE analysis showed the editing efficiency of crRNA1 (21.7%), crRNA2 (24.9%) and crRNA3 (55.6%) (**Figure 5**).

### 3.5 Confirmation of viral gene in plants and virus accumulation determination

**Figure 6:**
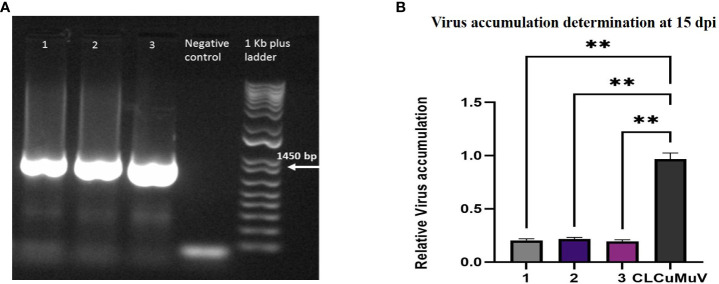
Confirmation of viral gene and virus accumulation determination in plants. **(A)** Lane 1–3 shows the presence of viral gene in plants infiltrated with Cas12a-MV and infectious clones. The master mix was used as a negative control. A 1KB Plus ladder was used. Primers were amplified at 1450 bp. **(B)** This graph represents the relative accumulation of CLCuMuV in *Nicotiana* plants at 15 dpi. Each bar (1–3) has three replicates of plants co-infiltered with Cas12a-MVconstruct and infectious clone (CLCuMuV). The plants showed low virus accumulation compared with the control (infectious clone). ** P<0.05 in ANOVA, which shows significant difference.

The presence of infectious clones in all plants was checked by PCR. Viral DNA was isolated from all infiltrated plants through the CTAB method and subjected to PCR using the virus specific primers CLVP1_500F and CLVP2_1500R. PCR confirmation of infectious clones in all infiltrated plants through PCR has been shown in **Figure 6A**. Amplified products of 1450bp with virus specific primers (CLVP1_500F and CLVP2_1500R) were resolved on 1% agarose gel and 1KB plus ladder was used as a marker. The virus accumulation in infected plants was determined by qPCR. The virus accumulation in *N. benthamiana* plants at 15dpi is shown in **Figure 6B**, which indicates the low virus accumulation (0.27) in plants containing Cas12a-MV as compared to the control plants (0.99), infiltrated with infectious clones.

### 3.6 Development of transgenic plant

**Figure 7:**
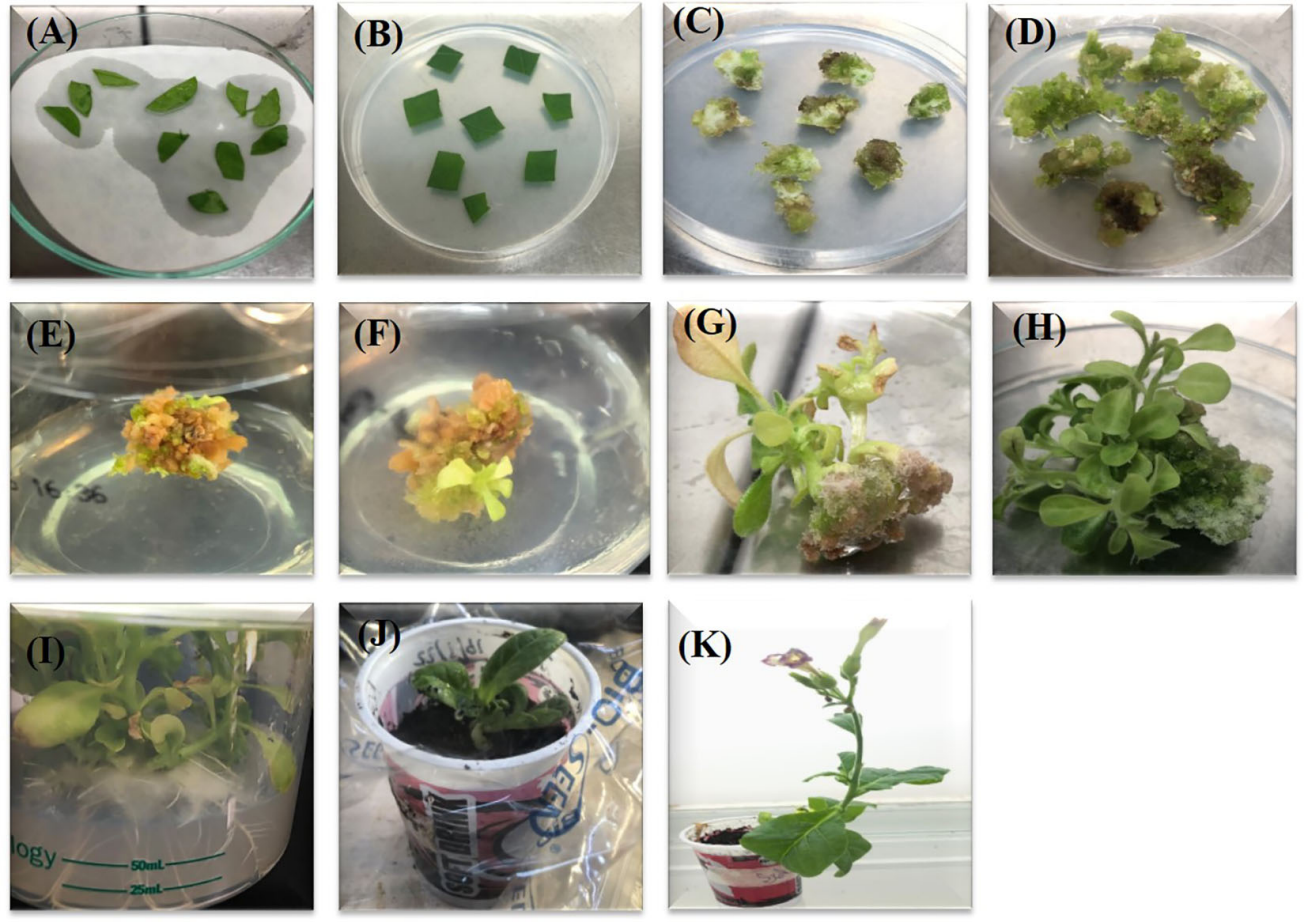
Different stages of *in-vitro* plant regeneration from leaf discs. **(A)** The inoculation (Agrobacterium suspension containing Cas12a-MV construct) of leaf discs (explant). **(B)** Shifting of inoculated explant to induction medium. **(C, D)** Transgenic Calli appeared within two weeks of transformation. **(E)** Regeneration of shoots from callus. **(F)** Initiation of shooting. **(G, H)** Shoots ready to shift on rooting media. **(I)** The rooting of putative transgenic shoots. **(J)** Transgenic plant under acclimatization conditions. **(K)** Transgenic plant.

Once Cas12a-MV construct was evaluated in *Nicotiana benthamiana* plants through transient transformation, the same construct was used for stable transformation in *Nicotiana tabacum* plants. About four-week-old plant leaf discs were infected with the agrobacterium containing Cas12a-MV construct (**Figure 7A**). The infected explants were placed on co-cultivation media and incubated at 22°C for two days to improve the transformation efficiency (**Figure 7B**). Explants (leaf discs) were placed on induction media and incubated at 28°C. Callus emerged from leaf discs after 1–2 weeks of transformation, as shown in **Figures 7C, D**. The transgenic callus was sub-cultured onto a freshly prepared induction medium after 7 days of interval, to avoid contamination (**Figure 7E**). Putative shoots were emerged from transgenic callus after four weeks (**Figure 7F**). Shoots of approximately 2–3 cm in length are shown in **Figures 7G, H**. Fully emerged shoots of 2–3 cm in size, were shifted to the rooting medium. Roots were developed in all transferred shoots (**Figure 7I**). All rooted plants were transferred to soil-filled small pots for acclimatization and kept under controlled conditions (**Figure 7J**). Three weeks old plants were moved to a greenhouse to collect seeds (**Figure 7K**).

### 3.7 Expression analysis of transgenic *Nicotiana* plants

**Figure 8:**
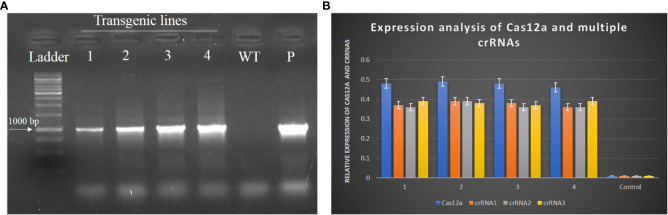
Evaluation of transgenic plants to check the expression of Cas12a and crRNAs. **(A)** PCR confirmed the presence of Cas12a in transgenic plants of *N. tabacum*. The size of the amplicon was 1KB. The purified vector was taken as the positive control and the DNA of wild-type plant was taken as the negative control. **(B)** Expression of Cas12a and multiple crRNAs was quantified through qPCR. All transgenic plants showed significant expression as compared to the control.

Transgenic *N. tabacum* plants were selected for transgene analyses. Genomic DNA was isolated from the leaves of transgenic *N. tabacum* plants through the CTAB method and subjected to PCR with specific primers of Cas12a. All plants showed amplification of the Cas12a gene, with amplicon of 1000bp in size, as shown in **Figure 8A**. The total RNA was isolated from all transgenic plants to evaluate the expression level of Cas12a and crRNAs through qPCR. The expression level of Cas12a (0.48) and all three crRNA1 (0.39), crRNA2 (0.39) and crRNA3 (0.38) are shown in **Figure 8B**. The actin gene of the *Nicotiana* plant was used as a control.

### 3.8 Transgenic plants with LbCas12a confer virus resistance

**Figure 9:**
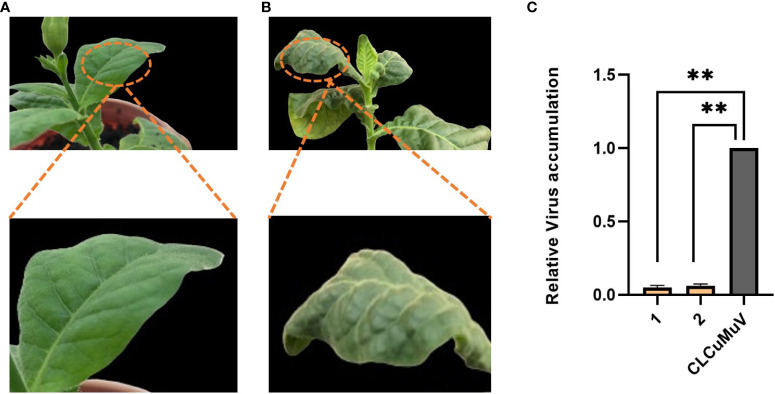
Evaluation of transgenic and wild-type plants of *N. tabacum* for CLCuMuV symptoms development. **(A)** The transgenic plant of *N. tabcum* was infiltrated with an infectious clone (CLCuMuV) and showed mild symptoms at early stages and no symptoms were observed at later stages and the plant became healthy. **(B)** Wild type plant of *N. tabacum* infiltrated with an infectious clone which showed severe symptoms. **(C)** This graph represents the relative accumulation of virus in transgenic plants of *N. tabacum* compared to control plants (only inoculated with CLCuMuV). Each bar (1–2) is the replicate of two transgenic plants. The transgenic plants showed the most promising results as they showed less virus accumulation (0.05) than the control plants (1.0) and remained healthy. ** P<0.05 in ANOVA, which shows significant difference.

The transgenic plants expressing the Cas12a-MV construct containing crRNAs showed rare symptoms after 7–8 dpi as shown in **Figure 9A**. The non-transgenic control plants showed leaf curl disease symptoms within 7–8 dpi as shown in **Figure 9B**. Virus accumulation in control (wild type inoculated with the infectious clone) and transgenic plants was determined through qPCR. It was observed that transgenic plants of *N. tabacum* (0.05) showed low virus accumulation compared to control plants (1.0), as shown in **Figure 9C**.

## 4 Discussion

### 4.1 Plant viruses and control strategies

Plant viruses infect most of the cash crops and seriously threaten food security in various countries. Chemical approach has been used to protect crops against insects, pests, fungal and bacterial infections, but these chemicals are ineffective against plant viruses (Bragard et al., 2013). Begomoviruses are a group of single-stranded DNA viruses that are known to infect plants mainly cotton, causing severe yield losses and economic consequences especially for the textile industry and farmers. CLCuV is the most devastating species of *Begomovirus* which has been responsible for the significant loss of cotton yield in Pakistan since the 1980s. There has been significant research on CLCuV, focused on understanding the biology of the virus and its interactions with the host plant, as well as the development of methods for controlling infections.

Many strategies such as crop rotation, chemical control and biotechnological methods have been used to control plant viruses and the most powerful one is pathogen-derived resistance (PDR). PDR is the process in which viral sequences are inserted in plant cells to produce resistance against virus. RNAi-mediated resistance in plants against plant viruses, is the type of PDR that has been widely used against RNA viruses, but this approach is ineffective for DNA viruses including begomoviruses. Sera, 2005 used artificial zinc finger protein to target the most conserved region (IR) of *Beet severe curly top virus* (BSCTV) and successfully suppressed the replication of BSCTV. Chen et al. (2014) evaluated the effect of zinc finger nucleases by targeting the *rep* gene of the *Tomato yellow leaf curl China virus* (TYLCCNV) and successfully achieved the mutations in the viral genome of TYLCCNV. Cheng et al. (2015) have demonstrated TALENs to engineer resistance against DNA viruses including TYLCCNV. Although ZFNs and TALEN have been demonstrated for developing virus resistance in plants, however, these genome editing tools have some limitations such as low efficiency, complex designing, expensive, laborious, targeting only a single site at a time and difficult to multiplex.

### 4.2 Potential applications of CRISPR/Cas system against plant viruses

CRISPR/Cas toolbox is a more robust alternative to generate resistance against plant viruses (Cao et al., 2020). CRISPR/Cas technology holds an excellent potential for improving crop productivity by developing resistance against biotic and abiotic stresses thus contributing to food security. Early gene editing studies with CRISPR/Cas9 were especially focused on agricultural improvement (Zhang et al., 2021). For example, CRISPR/Cas9 system has been successfully used to improve yield, disease resistance (Zhu et al., 2020), complex trait improvement and altering plant architecture (Bao et al., 2019). Different studies have specifically demonstrated the potential CRISPR/Cas9 toolbox to engineer resistance against plant viruses. Example includes, Tashkandi et al. (2018) used the CRISP/Cas9 system against the TYLCV in *benthimiana* and achieved the reduction in virus accumulation. Similarly, Liu et al. (2022) created the mutation in the *ZmGDIα* gene through Cas9 to increase the resistance against MRDD in maize plants. Multiplex CRISPR/Cas system has been suggested as a promising approach for controlling CLCuV and other plant viruses, as it allows researchers to target multiple viral genes simultaneously thus increasing the effectiveness of the treatment and reducing the risk of the viral escape. Multiplexing with LbCas12a offers a promising approach to target several genes simultaneously in the targeted organism.

In this study, we suppressed the replication of CLCuMuV through a multiplex Cas12a-based system in model plants named *Nicotiana benthamiana* and *Nicotiana tabacum*. Recent studies have demonstrated that targeting a viral genome at a single gene is not enough to limit viral replication and generates viral escape. McCarty et al. (2020) revealed that the potential solution for viral escape is targeting two or more genes simultaneously with multiple guide RNAs. In multiplexing, Cas nuclease and multiple guide RNAs are expressed simultaneously to target multiple genes (or regulatory sequences) to reduce the chances of viral escape. Kurata et al. (2018) demonstrated that the multiplex approach using more than one sgRNA to target multiple genes was more effective than the conventional single gene targeting methodology with CRISPR/Cas system.

Similarly, in the present study, LbCas12a nuclease and multiple crRNAs were expressed in the host plant, targeting the multiple genes of the viral genome. LbCas12a has been recommended for the editing of plants by Zhang and Qi (2021), who described it as an efficient system for gene modifications in rice. The authors also demonstrated the usefulness of the Cas12a system in generating plant mutants. In a similar research, Bernabé-Orts et al. (2019) used the Cas12a-based constructs for successful editing of the genomes of *Arabidopsis*, *benthimiana*, and *Lycopersicum*. The versatility of the Cas12a system has made it one of the favorite genome editing tools in life sciences with a broad range of applications. For example, Zhang et al. (2021) used the similar strategy to construct the multiplex Cas12a-based constructs through Golden Gateway cloning to target 16 sites simultaneously in rice. The authors achieved high editing efficiencies in rice with this multiplex CRISPR/Cas12a approach. All these findings demonstrate the excellent potential of multiplex Cas12a editing system in targeting multiple genes of plant genome simultaneously. In this study, we also successfully used the multiplex Cas12a vector to suppress the viral disease in model plants.

### 4.3 Sequence alignment

CRISPR/Cas system offers an excellent flexibility in terms of target selection. CLCuV is known for its high genetic variability, with numerous strains and variants circulating in different regions. By carefully selecting target sequences, conserved among different begomovirus strains, we can develop gRNAs with broad-spectrum activity against multiple virus variants. This would enable us to program the CRISPR/Cas-based strategies for an effective control of begomoviruses infections across diverse geographic locations. In this study, we aimed to investigate the geographic distribution of variants of the CLCuMuV species. To accomplish this, we collected genomic sequences of CLCuMuV variants, spanning the years 2019 to 2021 from the NCBI gene bank, and subsequently analyzed it on the Geneious Prime software. Multiple alignments of 92 CLCuMuV variants were performed using the Geneious Prime software to assess their similarity. The multiple alignments allowed us to compare the nucleotide sequences of all these variants and identify their conserved regions or potential variations present in their genomes. This analysis provided a valuable information regarding the genetic diversity within the CLCuMuV species. To evaluate the geographic distribution of the CLCuMuV variants, a phylogenetic tree was constructed using the Geneious Tree builder method within the Geneious Prime software. The phylogenetic tree provided insights into the evolutionary relationships and geographic clustering of these different CLCuMuV variants, enabling us to understand the spread and distribution patterns of this virus. We selected 27 CLCuMuV variants in Pakistan based on the geographic distribution analysis. Multiple alignments of these selected variants were conducted using Geneious Prime software to analyze their sequence conservation and variations. Comparative studies revealed interesting patterns between the selected CLCuMuV variants and those from other geographic regions. By comparing the genetic sequences and phylogenetic relationships, we observed similarities and differences among the CLCuMuV variants from Pakistan and other parts of the world. These findings revealed the relatedness and potential cross-contamination of CLCuMuV variants across different regions.

### 4.4 Designing of crRNAs for Cas12a: geneious prime software and other tools

One of the key advantages of the CRISPR/Cas system is its efficiency and specificity in targeting and modifying viral genomes. Naturally, Cas effectors, like Cas9 and Cas12, perform RNA-dependent DNA cleavage (Saito et al., 2023), which has been engineered for site specific genome editing in eukaryotes as well. A distinct advantage of RNA-guided systems is that it allows an effector to target multiple substrates by simply reprogramming the RNA guide (Altae-Tran et al., 2021) to different targets in the genome. By designing crRNAs complementary to the specific regions of the CLCuMuV genome, we induced targeted mutations in the viral genome, which resulted in the disruption of different viral genes, rendering the CLCuMuV unable to replicate and reduce the viral load in host plants. Geneious Prime software was used to design potential crRNAs (Doench et al., 2016). This software uses Python and R algorithms to find the CRISPR sites. Many other gRNA designing tools, including CHPCHOP, CRISPOR and CRISPR directs, have been used for designing gRNA, but these tools are only specified for Cas9 gRNA designing. Different Cas effectors and guide RNAs (gRNAs) design tools along with on target and off target parameters have been summarized by Li et al. (2022). However, Geneious Prime is a comprehensive bioinformatics software that provides a complete range to design gRNAs for the CRISPR/Cas9 and CRISPR/Cas12 systems. Geneious Prime is considered an excellent tool for gRNA design due to its fast and accurate algorithms, customizable parameters, and integration with other tools.

### 4.5 Cas12a-MV-mediated genome editing of CLCuMuV: comparative studies and insights

Our Cas12a-MV multiplex approach showed an efficient editing at specific sites of coding regions in CLCuMuV genome, leading to the suppression in the replication of CLCuMuV and viral load in the infiltrated plants. In this study, we found low virus accumulation in infiltrated plants with Cas12-MV construct, compared to control plants, infiltrated with infectious clones only. Real-time PCR was used to check the virus accumulation in infiltrated and control plants. The ct values obtained through real-time PCR were analyzed through one-way ANOVA. **Figure 6B** showed that the plants co-infiltrated with Cas12a-MV and infectious clones demonstrated less virus accumulation than control plants, which showed high virus accumulation. Binyameen et al. (2021) have also demonstrated a similar method that showed efficient suppression of CLCuKoV by simultaneously targeting multiple viral genes by multiplexed CRISPR/Cas9 thus reducing virus accumulation in host plants. In another study, Mubarik et al. (2021) also determined the virus accumulation in multiplex CRISPR/Cas9 transformed plants through real-time PCR and observed a 60–70% reduction in virus accumulation of CLCuKoV in transgenic plants. In another study, Khan et al. (2020) have demonstrated the reduced virus accumulation by 40–80% in *benthimiana* plants transformed with CRISPR/Cas9 against CLCuKoV. Similarly, Yin et al. (2019) used the CRISPR/Cas9 system to target and disrupt a specific region of the *rep* gene of CLCuMuV, which is known to be involved in the replication of the virus. By disrupting the *rep* gene, the researchers ultimately prevented the virus replication in transgenic *N. benthamiana* plants, conferring resistance to the virus. The researchers demonstrated that the edited plants were healthy and showed no disease symptoms compared with control plants.

In the present study, Cas12a-MV vector was stably transformed in *Nicotiana tabacum* plants to analyze the virus accumulation in transgenic *N. tabacum* plants. The virus accumulation in transgenic plants was determined by real-time PCR and compared with non-transgenic plants. Virus accumulation is an important parameter to assess the efficacy of the CRISPR/Cas system. The transgenic plants containing Cas12a-MV constructs showed lower virus accumulation compared with control plants. Severe symptoms were appeared in control plants, but transgenic plants remained healthy. Yin et al. (2019) also stably transformed the Cas9-based multiplex vector in *benthimiana* against CLCuMuV and achieved the resistance to viral infection, evaluated through Southern blotting in transgenic plants. In contrast, we have used real-time PCR to determine the virus accumulation. Real-time PCR is a highly sensitive and specific method that allows real-time detection and quantification of a particular sequence of DNA. It is a fast and an efficient technique that requires minimal sample preparation and can detect low levels of target DNA with high precision. On the other hand, Southern blotting is a time-consuming and labor-intensive technique that requires a relatively large amount of DNA and may not be as sensitive as real-time PCR.

### 4.6 Future prospects

CRISPR/Cas technology and its recent developments provide a rapid and efficient solution for controlling begomoviruses infections in crops. As this study was performed in model plants, further experiments are required to translate these results in cotton. The prospects for CRISPR/Cas technology against begomoviruses are promising, but it is not free of challenges and limitations. One of the critical challenges that need to be addressed is the durability of the resistance to begomoviruses. Viruses are known to evolve rapidly and can develop resistance to control measures. The efficacy of CRISPR/Cas-mediated resistance would also require continuous monitoring, and multiple approaches such as CRISPRi and CRISPR/Cas14, with new gRNAs targeting coding as well as non-coding regions in virus could be helpful to limit virus escape. Another challenge is, an efficient delivery of the CRISPR/Cas reagents into plant cells. New delivery methods with improved efficiency and specificity of different CRISPR reagents especially in cotton, are critical for the practical applications of CRISPR/Cas technology to improve cotton against viral and other stresses. In conclusion, the prospects of CRISPR/Cas technology against begomoviruses are positive, but further research is required to realize its full potential and translate results with model systems, in commercial crops. In addition, regulatory outcomes of CRISPR edited crops in different countries will also determine the future of these crops and their public acceptance.

## Data availability statement

The original contributions presented in the study are included in the article/**Supplementary Material**. Further inquiries can be directed to the corresponding authors.

## Author contributions

AA and SK designed the study. SA performed the experiments and wrote the manuscript. JB provided the experimental protocols and proofread the manuscript. AJ and BS revised the manuscript and gave suggestions to improve the article. All authors contributed to the article and approved the submitted version.

## References

[B1] Ali Z. Abulfaraj A. Idris A. Ali S. Tashkandi M. Mahfouz M. M. (2015). CRISPR/Cas9-mediated viral interference in plants. Genome Biol. 16, 1–11. doi: 10.1186/s13059-015-0799-6 26556628 PMC4641396

[B2] Al Shihi A. A. M. (2019). Geminivirus occurrence in Australia, China, Europe, and the Middle Eastern countries. Geminiviruses: Impact Challenges Approaches, 65–83. doi: 10.1007/978-3-030-18248-9_4

[B3] Altae-Tran H. Kannan S. Demircioglu F. E. Oshiro R. Nety S. P. McKay L. J.. (2021). The widespread IS200/IS605 transposon family encodes diverse programmable RNA-guided endonucleases. Science 374 (6563), 57–65. doi: 10.1126/science.abj6856 34591643 PMC8929163

[B4] Bae S. Park J. Kim J. S. (2014). Cas-OFFinder: a fast and versatile algorithm that searches for potential off-target sites of Cas9 RNA-guided endonucleases. Bioinformatics 30 (10), 1473–1475. doi: 10.1093/bioinformatics/btu048 24463181 PMC4016707

[B5] Baltes N. J. Hummel A. W. Konecna E. Cegan R. Bruns A. N. Bisaro D. M.. (2015). Conferring resistance to geminiviruses with the CRISPR–Cas prokaryotic immune system. Nat. Plants 1 (10), 1–4. doi: 10.1038/nplants.2015.145 PMC8612103 34824864

[B6] Bananej K. Kraberger S. Varsani A. (2016). Okra enation leaf curl virus in papaya from Iran displaying severe leaf curl symptoms. J. Plant Pathol. 98 (3), 637–639.

[B7] Bandyopadhyay A. Kancharla N. Javalkote V. S. Dasgupta S. Brutnell T. P. (2020). CRISPR-Cas12a (Cpf1): a versatile tool in the plant genome editing tool box for agricultural advancement. Front. Plant Sci. 11. doi: 10.3389/fpls.2020.584151 PMC7668199 33214794

[B8] Bao A. Chen H. Chen L. Chen S. Hao Q. Guo W.. (2019). CRISPR/Cas9-mediated targeted mutagenesis of GmSPL9 genes alters plant architecture in soybean. BMC Plant Biol. 19 (1), 1–12. doi: 10.1186/s12870-019-1746-6 30961525 PMC6454688

[B9] Bernabé-Orts J. M. Casas-Rodrigo I. Minguet E. G. Landolfi V. Garcia-Carpintero V. Gianoglio S.. (2019). Assessment of Cas12a-mediated gene editing efficiency in plants. Plant Biotechnol. J. 17 (10), 1971–1984. doi: 10.1111/pbi.13113 30950179 PMC6737022

[B10] Binyameen B. Khan Z. Khan S. H. Ahmad A. Munawar N. Mubarik M. S.. (2021). Using multiplexed CRISPR/Cas9 for suppression of cotton leaf curl virus. Int. J. Mol. Sci. 22 (22), 12543. doi: 10.3390/ijms222212543 34830426 PMC8618328

[B11] Bragard C. Caciagli P. Lemaire O. Lopez-Moya J. MacFarlane S. Peters D.. (2013). Status and prospects of plant virus control through interference with vector transmission. Annu. Rev. Phytopathol. 51, 177–201. doi: 10.1146/annurev-phyto-082712-102346 23663003

[B12] Brinkman E. K. van Steensel B. (2019). Rapid quantitative evaluation of crispr genome editing by tide and tider. CRISPR Gene Editing: Methods Protoc. 1961, 29–44. doi: 10.1007/978-1-4939-9170-9_3 30912038

[B13] Cao Y. Zhou H. Zhou X. Li F. (2020). Control of plant viruses by CRISPR/Cas system-mediated adaptive immunity. Front. Microbiol. 11. doi: 10.3389/fmicb.2020.593700 PMC7649272 33193268

[B14] Caplan A. L. Parent B. Shen M. Plunkett C. (2015). No time to waste—the ethical challenges created by CRISPR: CRISPR/Cas, being an efficient, simple, and cheap technology to edit the genome of any organism, raises many ethical and regulatory issues beyond the use to manipulate human germ line cells. EMBO Rep. 16 (11), 1421–1426. doi: 10.15252/embr.201541337 26450575 PMC4641494

[B15] Chaparro-Garcia A. Kamoun S. Nekrasov V. (2015). Boosting plant immunity with CRISPR/Cas. Genome Biol. 16 (1), 1–4. doi: 10.1186/s13059-015-0829-4 26585913 PMC4653885

[B16] Chen W. Qian Y. Wu X. Sun Y. Wu X. Cheng X. (2014). Inhibiting replication of begomoviruses using artificial zinc finger nucleases that target viral-conserved nucleotide motif. Virus Genes 48, 494–501. doi: 10.1007/s11262-014-1041-4 24474330

[B17] Cheng X. Li F. Cai J. Chen W. Zhao N. Sun Y.. (2015). Artificial TALE as a convenient protein platform for engineering broad-spectrum resistance to begomoviruses. Viruses 7 (8), 4772–4782. doi: 10.3390/v7082843 26308041 PMC4576204

[B18] Dethier J.-J. Effenberger A. (2012). Agriculture and development: A brief review of the literature. Economic Syst. 36 (2), 175–205. doi: 10.1016/j.ecosys.2011.09.003

[B19] Doench J. G. Fusi N. Sullender M. Hegde M. Vaimberg E. W. Donovan K. F.. (2016). Optimized sgRNA design to maximize activity and minimize off-target effects of CRISPR-Cas9. Nat. Biotechnol. 34 (2), 184–191. doi: 10.1038/nbt.3437 26780180 PMC4744125

[B20] Farooq J. Farooq A. Riaz M. Shahid M. Saeed F. Iqbal M.. (2014). Cotton leaf curl virus disease a principle cause of decline in cotton productivity in Pakistan (a mini review). Can. J. Plant Prot 2, 9–16.

[B21] Fellmann C. Gowen B. G. Lin P.-C. Doudna J. A. Corn J. E. (2017). Cornerstones of CRISPR–Cas in drug discovery and therapy. Nat. Rev. Drug Discovery 16 (2), 89–100. doi: 10.1038/nrd.2016.238 28008168 PMC5459481

[B22] Franz A. Brunner E. Basler K. (2017). Generation of genome-modified Drosophila cell lines using SwAP. Fly 11 (4), 303–311. doi: 10.1080/19336934.2017.1372068 28853976 PMC5721941

[B23] Gaj T. Gersbach C. A. Barbas C. F. (2013). ZFN, TALEN, and CRISPR/Cas-based methods for genome engineering. Trends Biotechnol. 31 (7), 397–405. doi: 10.1016/j.tibtech.2013.04.004 23664777 PMC3694601

[B24] Gao C. (2021). Genome engineering for crop improvement and future agriculture. Cell 184 (6), 1621–1635. doi: 10.1016/j.cell.2021.01.005 33581057

[B25] Gosavi G. Yan F. Ren B. Kuang Y. Yan D. Zhou X.. (2020). Applications of CRISPR technology in studying plant-pathogen interactions: overview and perspective. Phytopathol. Res. 2 (1), 1–9. doi: 10.1186/s42483-020-00060-z

[B26] Healey A. Furtado A. Cooper T. Henry R. J. (2014). Protocol: a simple method for extracting next-generation sequencing quality genomic DNA from recalcitrant plant species. Plant Methods 10 (1), 1–8. doi: 10.1186/1746-4811-10-21 25053969 PMC4105509

[B27] Hameed A. Zaidi S.S.-E.-A. Sattar M. N. Iqbal Z. Tahir M. N. (2019). CRISPR technology to combat plant RNA viruses: A theoretical model for Potato virus Y (PVY) resistance. Microbial pathogenesis 133, 103551. doi: 10.1016/j.micpath.2019.103551 31125685

[B28] Hameed U. Zia-Ur-Rehman M. Herrmann H.-W. Haider M. Brown J. (2014). First report of Okra enation leaf curl virus and associated cotton leaf curl Multan betasatellite and cotton leaf curl Multan alphasatellite infecting cotton in Pakistan: a new member of the cotton leaf curl disease complex. Plant Dis. 98 (10), 1447–1447. doi: 10.1094/PDIS-04-14-0345-PDN 30704006

[B29] Jameel M. R. Ansari Z. Qureshi M. I. (2022). From design to validation of CRISPR/gRNA primers towards genome editing. Bioinformation 18 (5), 471–477. doi: 10.6026/97320630018471 36945226 PMC10024777

[B30] Khan S. Mahmood M. Rahman S. Rizvi F. Ahmad A. (2020). Evaluation of the CRISPR/Cas9 system for the development of resistance against Cotton leaf curl virus in model plants. Plant Prot. Sci. 56 (3), 154–162. doi: 10.17221/105/2019-PPS

[B31] Kil E.-J. Kim S. Lee Y.-J. Byun H.-S. Park J. Seo H.. (2016). Tomato yellow leaf curl virus (TYLCV-IL): a seed-transmissible geminivirus in tomatoes. Sci. Rep. 6 (1), 19013. doi: 10.1038/srep19013 26743765 PMC4705557

[B32] Kothandaraman S. V. Devadason A. Ganesan M. V. (2016). Seed-borne nature of a begomovirus, Mung bean yellow mosaic virus in black gram. Appl. Microbiol. Biotechnol. 100, 1925–1933. doi: 10.1007/s00253-015-7188-7 26646557

[B33] Kurata M. Wolf N. K. Lahr W. S. Weg M. T. Kluesner M. G. Lee S.. (2018). Highly multiplexed genome engineering using CRISPR/Cas9 gRNA arrays. PloS One 13 (9), e0198714. doi: 10.1371/journal.pone.0198714 30222773 PMC6141065

[B34] Li C. Chu W. Gill R. A. Sang S. Shi Y. Hu X.. (2022). Computational tools and resources for CRISPR/Cas genome editing. Genomics Proteomics Bioinf. 21, 108–126. doi: 10.1016/j.gpb.2022.02.006 PMC10372911 35341983

[B35] Liu C. Kong M. Yang F. Zhu J. Qi X. Weng J.. (2022). Targeted generation of Null Mutants in ZmGDIα confers resistance against maize rough dwarf disease without agronomic penalty. Plant Biotechnol. J. 20 (5), 803. doi: 10.1111/pbi.13793 35178853 PMC9055807

[B36] Ma X. Zhang Q. Zhu Q. Liu W. Chen Y. Qiu R.. (2015). A robust CRISPR/Cas9 system for convenient, high-efficiency multiplex genome editing in monocot and dicot plants. Mol. Plant 8 (8), 1274–1284. doi: 10.1016/j.molp.2015.04.007 25917172

[B37] Ma X. Zhu Q. Chen Y. Liu Y.-G. (2016). CRISPR/Cas9 platforms for genome editing in plants: developments and applications. Mol. Plant 9 (7), 961–974. doi: 10.1016/j.molp.2016.04.009 27108381

[B38] Makkouk K. M. (2020). Plant pathogens which threaten food security: Viruses of chickpea and other cool season legumes in West Asia and North Africa. Food Secur. 12 (3), 495–502. doi: 10.1007/s12571-020-01017-y

[B39] Malzahn A. Lowder L. Qi Y. (2017). Plant genome editing with TALEN and CRISPR. Cell bioscience 7 (1), 1–18. doi: 10.1186/s13578-017-0148-4 28451378 PMC5404292

[B40] Matthews R. E. F. (2012). Plant virology (San Diego, California: Elsevier).

[B41] McCarty N. S. Graham A. E. Studená L. Ledesma-Amaro R. (2020). Multiplexed CRISPR technologies for gene editing and transcriptional regulation. Nat. Commun. 11 (1), 1281. doi: 10.1038/s41467-020-15053-x 32152313 PMC7062760

[B42] Mishra R. Zheng W. Joshi R. K. Kaijun Z. (2021). Genome editing strategies towards enhancement of rice disease resistance. Rice Sci. 28 (2), 133–145. doi: 10.1016/j.rsci.2021.01.003

[B43] Mubarik M. S. Khan S. H. Sadia B. Ahmad A. (2019). CRISPR-Cas9 based suppression of cotton leaf curl virus in Nicotiana benthamina. Int. J. Agric. Biol. 22, 517–522. doi: 10.17957/IJAB/15.1094

[B44] Mubarik M. S. Wang X. Khan S. H. Ahmad A. Khan Z. Amjid M. W.. (2021). Engineering broad-spectrum resistance to cotton leaf curl disease by CRISPR-Cas9 based multiplex editing in plants. GM Crops Food 12 (2), 647–658. doi: 10.1080/21645698.2021.1938488 34124996 PMC9208622

[B45] Panattoni A. Luvisi A. Triolo E. (2013). Elimination of viruses in plants: twenty years of progress. Spanish J. Agric. Res. 1), 173–188. doi: 10.5424/sjar/2013111-3201

[B46] Rahman M.-u. Zulfiqar S. Raza M. A. Ahmad N. Zhang B. (2022). Engineering abiotic stress tolerance in crop plants through CRISPR genome editing. Cells 11 (22), 3590. doi: 10.3390/cells11223590 36429019 PMC9688763

[B47] Ramesh S. V. Shivakumar M. Ramteke R. Bhatia V. S. Chouhan B. S. Goyal S.. (2019). Quantification of a legume begomovirus to evaluate soybean genotypes for resistance to yellow mosaic disease. J. virological Methods 268, 24–31. doi: 10.1016/j.jviromet.2019.03.002 30890330

[B48] Rehman A. Jingdong L. Chandio A. A. Hussain I. Wagan S. A. Memon Q. U. A. (2019). Economic perspectives of cotton crop in Pakistan: A time series analysis, (1970–2015)(Part 1). J. Saudi Soc. Agric. Sci. 18 (1), 49–54. doi: 10.1016/j.jssas.2016.12.005

[B49] Rosen R. Kanakala S. Kliot A. Pakkianathan B. C. Farich B. A. Santana-Magal N.. (2015). Persistent, circulative transmission of begomoviruses by whitefly vectors. Curr. Opin. Virol. 15, 1–8. doi: 10.1016/j.coviro.2015.06.008 26196230

[B50] Saito M. Xu P. Faure G. Maguire S. Kannan S. Altae-Tran H.. (2023). Fanzor is a eukaryotic programmable RNA-guided endonuclease. Nature, 1–3. doi: 10.1038/s41586-023-06356-2 PMC10432273 37380027

[B51] Sattar M. N. Kvarnheden A. Saeed M. Briddon R. W. (2013). Cotton leaf curl disease–an emerging threat to cotton production worldwide. J. Gen. Virol. 94 (4), 695–710. doi: 10.1099/vir.0.049627-0 23324471

[B52] Sera T. (2005). Inhibition of virus DNA replication by artificial zinc finger proteins. J. Virol. 79 (4), 2614–2619. doi: 10.1128/jvi.79.4.2614-2619.2005 15681461 PMC546585

[B53] Shan Q. Wang Y. Li J. Gao C. (2014). Genome editing in rice and wheat using the CRISPR/Cas system. Nat. Protoc. 9 (10), 2395–2410. doi: 10.1038/nprot.2014.157 25232936

[B54] Sumner D. R. (2018). “Crop rotation and plant productivity,” in CRC handbook of agricultural productivity (Boca Raton: CRC Press), 273–314.

[B55] Swarts D. C. (2019). Making the cut (s): how Cas12a cleaves target and non-target DNA. Biochem. Soc. Trans. 47 (5), 1499–1510. doi: 10.1042/BST20190564 31671185

[B56] Tashkandi M. Ali Z. Aljedaani F. Shami A. Mahfouz M. M. (2018). Engineering resistance against Tomato yellow leaf curl virus *via* the CRISPR/Cas9 system in tomato. Plant Signaling Behav. 13 (10), e1525996. doi: 10.1080/15592324.2018.1525996 PMC6204811 30289378

[B57] Thakur H. Jindal S. K. Sharma A. Dhaliwal M. S. (2018). Chilli leaf curl virus disease: a serious threat for chilli cultivation. J. Plant Dis. Prot. 125, 239–249. doi: 10.1007/s41348-018-0146-8

[B58] Tzean Y. Lee M.-C. Jan H.-H. Chiu Y.-S. Tu T.-C. Hou B.-H.. (2019). Cucumber mosaic virus-induced gene silencing in banana. Sci. Rep. 9 (1), 1–9. doi: 10.1038/s41598-019-47962-3 31399618 PMC6689018

[B59] Uniyal A. P. Yadav S. K. Kumar V. (2019). The CRISPR–Cas9, genome editing approach: a promising tool for drafting defense strategy against begomoviruses including cotton leaf curl viruses. J. Plant Biochem. Biotechnol. 28 (2), 121–132. doi: 10.1007/s13562-019-00491-6

[B60] Yin K. Han T. Xie K. Zhao J. Song J. Liu Y. (2019). Engineer complete resistance to Cotton Leaf Curl Multan virus by the CRISPR/Cas9 system in Nicotiana benthamiana. Phytopathol. Res. 1 (1), 1–9. doi: 10.1186/s42483-019-0017-7

[B61] Yu Y. Wang X. Sun H. Liang Q. Wang W. Zhang C.. (2020). Improving CRISPR-Cas-mediated RNA targeting and gene editing using SPLCV replicon-based expression vectors in Nicotiana benthamiana. Plant Biotechnol. J. 18 (10), 1993. doi: 10.1111/pbi.13384 32289196 PMC7539982

[B62] Zafar S. A. Zaidi S.S.-E.-A. Gaba Y. Singla-Pareek S. L. Dhankher O. P. Li X.. (2020). Engineering abiotic stress tolerance *via* CRISPR/Cas-mediated genome editing. J. Exp. Bot. 71 (2), 470–479. doi: 10.1093/jxb/erz476 31644801

[B63] Zaidi S.-e. Mansoor S. Ali Z. Tashkandi M. Mahfouz M. M. (2016). Engineering plants for geminivirus resistance with CRISPR/Cas9 system. Trends Plant Sci. 21 (4), 279–281. doi: 10.1016/j.tplants.2016.01.023 26880316

[B64] Zerbini F. M. Briddon R. W. Idris A. Martin D. P. Moriones E. Navas-Castillo J.. (2017). ICTV virus taxonomy profile: Geminiviridae. J. Gen. Virol. 98 (2), 131. doi: 10.1099/jgv.0.000738 28284245 PMC5802298

[B65] Zhang Y. Malzahn A. A. Sretenovic S. Qi Y. (2019). The emerging and uncultivated potential of CRISPR technology in plant science. Nat. Plants 5 (8), 778–794. doi: 10.1038/s41477-019-0461-5 31308503

[B66] Zhang Y. Qi Y. (2021). “Efficient multiplexed CRISPR-Cas12a genome editing in plants,” in CRISPR-Cas Methods, vol. 2. (Humana, New York, NY: Springer), 41–56.

[B67] Zhang Y. Ren Q. Tang X. Liu S. Malzahn A. A. Zhou J.. (2021). Expanding the scope of plant genome engineering with Cas12a orthologs and highly multiplexable editing systems. Nat. Commun. 12 (1), 1944. doi: 10.1038/s41467-021-22330-w 33782402 PMC8007695

[B68] Zhang T. Zheng Q. Yi X. An H. Zhao Y. Ma S.. (2018). Establishing RNA virus resistance in plants by harnessing CRISPR immune system. Plant Biotechnol. J. 16 (8), 1415–1423. doi: 10.1111/pbi.12881 29327438 PMC6041442

[B69] Zhu H. Li C. Gao C. (2020). Applications of CRISPR–Cas in agriculture and plant biotechnology. Nat. Rev. Mol. Cell Biol. 21 (11), 661–677. doi: 10.1038/s41580-020-00288-9 32973356

[B70] Zubair M. Zaidi S.S.-E.-A. Shakir S. Amin I. Mansoor S. (2017a). An insight into Cotton leaf curl Multan betasatellite, the most important component of cotton leaf curl disease complex. Viruses 9 (10), 280. doi: 10.3390/v9100280 28961220 PMC5691632

[B71] Zubair M. Zaidi S.S.-E.-A. Shakir S. Farooq M. Amin I. Scheffler J. A.. (2017b). Multiple begomoviruses found associated with cotton leaf curl disease in Pakistan in early 1990 are back in cultivated cotton. Sci. Rep. 7 (1), 680. doi: 10.1038/s41598-017-00727-2 28386113 PMC5429635

