# Supplementary material for: LbCas12a mediated suppression of Cotton leaf curl Multan virus
Source: Front Plant Sci. 2023 Aug 11;14:1233295. doi: 10.3389/fpls.2023.1233295 (PMC10456881; doi:10.3389/fpls.2023.1233295)
Supplement: Supplementary file 1 [file Table_1.docx]

Supplementary Material

# Supplementary Figures and Tables

**Table S1** shows the list of primers which was used for whole genome sequencing of cloned construct Cas12-MV.

| - **Primer** | - **Name** | - **Sequence (5’→3’)** |
| --- | --- | --- |
| - Forward Primer | - Ubi-intron-F1 | - CCCTGTTGTTTGGTGTTACTTC |
| - Reverse Primer | - M13-R1 | - TTTGAGACACGGGCCAGAGCTGC |

**Table S2** shows the List of primers used to amplify infectious clone sequence.

| **Primer** | **Name of primer** | **Sequence (5’→3’)** |
| --- | --- | --- |
| Forward Primer | CLVP1_500F | GGATGTACAGAAGTCCRGATGTTCC |
| Reverse Primer | CLVP2_1500R | CAACCATGGATTCWCGCACAGGGGAAC |

**Table S3** shows the list of primers which was used for Sanger sequencing.

| **No.** | **Primer name** | **Primer Sequence (5’→3’)** |
| --- | --- | --- |
| 1 | CLCuMuV-F1 | TCGGAGACCCACTCTTCAAG |
|  | CLCuMuV-R1 | AAACCCGTGAACCGTATC |
| 2 | CLCuMuV-F2 | GATAATGAGCCCAGTACGGC |
|  | CLCuMuV-R2 | CTGACAATCAACTGCCAC |

**Table S4** Selected Pakistani CLCuMuV species with their accession numbers retrieved through NCBI

| **No.** | **Name** | **Accession Number** | **Size (bp)** | **Origin (Pakistan)** |
| --- | --- | --- | --- | --- |
| 1 | CLCuMuV isolate NAS-85 | MT037028.1 | 2738 | Rajasthan |
| 2 | CLCuMuV isolate NAS-86 | MT037029.1 | 2738 | Rajasthan |
| 3 | CLCuMuV isolate NAS-87 | MT037030.1 | 2738 | Rajasthan |
| 4 | CLCuMuV isolate NAS-88 | MT037031.1 | 2738 | Rajasthan |
| 5 | CLCuMuV isolate NAS-89 | MT037032.1 | 2738 | Rajasthan |
| 6 | CLCuMuV isolate NAS-90 | MT037033.1 | 2738 | Rajasthan |
| 7 | CLCuMuV isolate AK-10 | MT966797.1 | 2738 | Rajasthan |
| 8 | CLCuMuV isolate AK-11 | MT966798.1 | 2738 | Punjab |
| 9 | CLCuMuV isolate AK-12 | MT966799.1 | 2738 | Punjab |
| 10 | CLCuMuV isolate AK-13 | MT966800.1 | 2738 | Punjab |
| 11 | CLCuMuV isolate AK-14 | MT966801.1 | 2738 | Punjab |
| 12 | CLCuMuV isolate AK-15 | MT966802.1 | 2738 | Punjab |
| 13 | CLCuMuV isolate NAS-1 | MK357244.1 | 2738 | Punjab |
| 14 | CLCuMuV isolate NAS-2 | MK357245.1 | 2738 | Punjab |
| 15 | CLCuMuV isolate NAS-3 | MK357246.1 | 2738 | Punjab |
| 16 | CLCuMuV isolate NAS-4 | MK357247.1 | 2738 | Punjab |
| 17 | CLCuMuV isolate NAS-5 | MK357248.1 | 2738 | Punjab |
| 18 | CLCuMuV isolate NAS-6 | MK357249.1 | 2738 | Punjab |
| 19 | CLCuMuV isolate NAS-7 | MK357250.1 | 2738 | Punjab |
| 20 | CLCuMuV isolate NAS-8 | MK357251.1 | 2738 | Punjab |
| 21 | CLCuMuV isolate NAS-9 | MK357252.1 | 2738 | Punjab |
| 22 | CLCuMuV isolate NAS-10 | MK357253.1 | 2738 | Punjab |
| 23 | CLCuMuV isolate NAS-11 | MK357254.1 | 2738 | Punjab |
| 24 | CLCuMuV isolate NAS-12 | MK357255.1 | 2738 | Punjab |
| 25 | CLCuMuV isolate NAS-13 | MK357256.1 | 2738 | Punjab |
| 26 | CLCuMuV isolate NAS-14 | MK357257.1 | 2738 | Punjab |
| 27 | CLCuMuV isolate NAS-15 | MK357258.1 | 2738 | Punjab |

**Table S5** Different properties of selected crRNAs including secondary structure, hairpin structure, self-dimerization, heterodimerization and off-target effects.

| **Name** | **Target region** | **PAM Sequence** | **Sequence** | **Position** | **Strand** | **Hairpin** | **Self-dimerization** | **Heterodimerization** | **Off-target effect in *N.benthimiana*/Cotton** |
| --- | --- | --- | --- | --- | --- | --- | --- | --- | --- |
| crRNA1 | C1 | TTTA | GGAGCTAGTTCCTTAATGAC | 2119 to 2142 | +IVE | -1.59 | -6.34 | -2.91 | No |
| crRNA2 | C2 and C3 | TTTC | TAACCTTCCGAATCTGGACG | 1258 to 1235 | -IVE | -0.49 | -4.64 | -3.61 | No |
| crRNA3 | V1 | TTTC | CGTATGATTCTCGTATTTTC | 948 to 925 | -IVE | 24.8 | -3.61 | -7.39 | No |

**
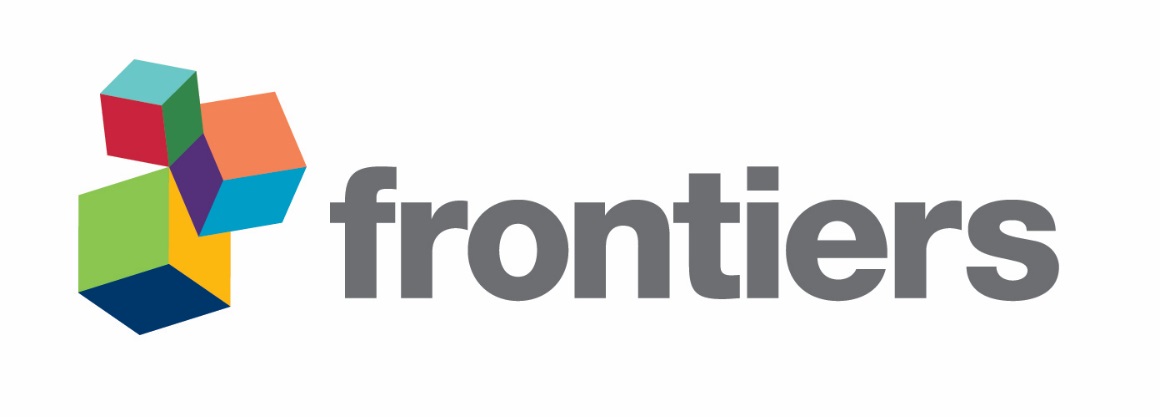
**
